# Beverage Intake among Children: Associations with Parent and Home-Related Factors

**DOI:** 10.3390/ijerph14080929

**Published:** 2017-08-18

**Authors:** Arwa Zahid, Cynthia Davey, Marla Reicks

**Affiliations:** 1 Department of Food Science and Nutrition, University of Minnesota, Minneapolis, MN 55455, USA; zahid001@umn.edu; 2 Clinical and Translational Science Institute, University of Minnesota, Minneapolis, MN 55455, USA; davey002@umn.edu

**Keywords:** children, beverage intake, parenting practices, nutrition knowledge

## Abstract

Beverage intake can influence child diet quality in a positive or negative manner depending on the beverage type and amounts consumed. Parenting practices such as role modeling and control of home beverage availability have been associated with child beverage intake, whereas examination of the influence of parental beverage nutrition knowledge has been more limited. The purpose of this study was to examine the relationships between sugar-sweetened and dairy beverage intake among children (9–12 years) and home and parental factors. A questionnaire was administered among a convenience sample of parents (*n* = 194) to assess beverage nutrition knowledge, beverage intake and home availability of beverages. Children completed a questionnaire to estimate usual beverage intake. Daily sugar-sweetened beverage intake by children ranged from 0.4 to 48 oz. Logistic regression analysis was used to examine relationships. Parents were mostly female, white, well educated, and employed. Home availability of sugar-sweetened and dairy beverages was positively associated with child sugar-sweetened (OR = 1.48, *p* = 0.03) and dairy beverage intake (OR = 1.34, *p* = 0.03), respectively. Parent dairy beverage intake was associated with child dairy beverage intake (OR = 1.06, *p* = 0.01). Parent knowledge about sugar in beverages was related to child dairy beverage intake (OR = 1.46, *p* = 0.02), whereas calcium/dairy knowledge and general beverage nutrition knowledge were not related to child beverage intake. Parenting practices and knowledge may play a role in determining child beverage intake.

## 1. Introduction

Beverages play a major role in the diets of children and adolescents [1]. Sugar-sweetened beverages (SSBs) are a leading source of empty calories and are considered one of the key elements of child obesity prevention initiatives in the United States (U.S.) [1]. On the other hand, milk provides important nutrients such as protein, calcium and vitamin D in addition to calories [2]. 

Intake of SSBs by U.S. children and adolescents is a concern because of associated health issues [3,4,5,6]. Data from the National Health and Nutrition Examination Survey (NHANES) 2011–2014 showed that nearly two-thirds of U.S. youth (2–19 years) consumed at least one SSB on a given day [7]. Beverages accounted for 47% of added sugars in the diet for children and adolescents (NHANES 2009–2010) [1]. Poor diet quality in children was linked with beverage consumption patterns high in sugars and low in dairy beverages [8,9]. Three meta-analyses showed relationships between SSB intake and weight gain and type 2 diabetes in children and adolescents [4,5,10]. 

Intake of dairy foods is associated with meeting nutrient intake requirements for growth and development for children and adolescents [2]. However, many adolescents (9–18 years) in the U.S. do not meet 2010 Dietary Guidelines recommendations for intake of 3 daily servings of dairy products (99% of girls and 78% of boys) based on NHANES 2007–2010 data [11]. Over the past several decades, milk intake has decreased among children and adolescents [2,12], making it less likely that recommendations for dairy food intake are being met.

Using Social Cognitive Theory as an organizing framework [13], parenting practices such as role modeling and controlling home beverage availability are considered part of the social and physical environment which can be manipulated to change beverage intake behaviors of children [14,15]. Evidence for a relationship between parenting practices and SSB intake among children and adolescents [16,17,18] is more extensive than the limited number of studies examining the role that parenting practices play in influencing intake of dairy beverages [19,20,21,22,23]. 

Parental influence on child beverage intake may be related to knowledge about diet and health. SSB intake among adults was significantly associated with knowledge about SSBs [24], thus potentially affecting the potential to role model intake for children. However, few studies have examined the role of parental nutrition or beverage knowledge on child beverage intake or on parenting practices that could affect intake. In one study, parental nutrition knowledge (including knowledge about SSBs) was a significant predictor of Norwegian adolescents’ nutrition knowledge [25]. However, SSB intake among adolescents was not significantly related to either parental or adolescent knowledge scores. In another study, parents of children 2 to 17 years (80% women, 54% White) perceived that sugary drinks, specifically sport drinks, fruit drinks, and flavored waters, were healthy options for their children [26]. Interviews with 201 parents of early adolescent children showed limited knowledge about calcium functions, requirements, and food sources and expectations for regular consumption of calcium-rich foods by children [27]. The lack of previous studies and inconsistent results of existing studies indicates a need to better characterize the relationship between parental knowledge and beverage intake of children.

Parental influence on beverage decision making and behaviors needs to be addressed during the critical developmental period of early adolescence as dietary behaviors tend to track into adulthood [21]. The purpose of this study was to test the hypotheses that associations exist between beverage intakes among early adolescent children (9–12 years) and home and parental factors such as knowledge regarding beverages, beverage home availability, and role modeling beverage intake.

## 2. Materials and Methods 

### 2.1. Participants

A convenience sample of parents/caregivers (*n* = 194) completed a questionnaire at the Minnesota State Fair in 2014 in a building specifically designed for research studies (Driven to Discover building). Parents were recruited for the study with signage posted in the building. They were eligible if they were the person primarily responsible for food acquisition/preparation of a child (9–12 years), and could complete a questionnaire in English. Children of parent participants were eligible if they were 9–12 years of age. The University Institutional Review Board approved the study with consent and assent procedures (IRB Code Number: 1405P50922). Parents and children were each given $5 in State Fair ride tickets as compensation for their participation.

### 2.2. Parent Questionnaire

Items were created to assess knowledge about healthful and less healthful beverages with respect to composition (energy, nutrients, and portion sizes), recommended intakes, and relationship to health [1,28,29,30]. Several University faculty and graduate students reviewed the items for content validity. Nutrition undergraduate and graduate students (*n* = 97) answered the questions to assess the difficulty level and provided comments about comprehension. Items were revised as needed based on these responses. After revision, items were organized into three main categories including beverage nutrition knowledge (8 items), dairy/calcium knowledge (8 items), and knowledge about sugar in beverages (7 items). To score the items, each correct answer was assigned a point value of 1 with a possible score ranging from 0–8 or 0–7 depending on the number of items (See Supplementary Materials).

Home availability of various beverage types was assessed using 9 questions previously evaluated for reliability and validity with parents of adolescents [31]. The questions asked: “How often would you say these beverages are available in your home?” Beverages included milk, soft drinks, fruit drinks, fruit juice, and water and the response options included 1 = always–4 = never. Items were grouped and summed to construct continuous variables to assess the availability of SSBs (regular soda pop and fruit drinks) and dairy-based beverages (whole milk, 1%, 2% or soy milk; flavored milk; blended yogurt and juice drink or yogurt drink).

A previously validated 15-item beverage questionnaire was used to assess usual beverage intake among parents as an indication of modeling beverage intakes for children [32]. Beverage items included soft drinks, dairy beverages, fruit juice, water, caffeinated, and energy beverages. Respondents were asked to indicate their usual intake over the past month by indicating how often they consumed the beverage (never or less than 1 per week, 1/week, 2–3/week, 4–6/week, 1/day, 2+/day, 3+/day) and how much they consumed (less than 6 oz, 8 oz, 12 oz, more than 12 oz). Children completed 9 questions about the frequency of beverage intake from the Harvard food frequency questionnaire to assess usual intake with added questions about specific beverages [33]. The beverages were soda pop, fruit-flavored drinks, fruit juice, café latte, coffee or tea, cocoa, milk, milk on cereal, and water.

Items were grouped and summed to construct continuous variables to assess daily intake of SSBs. For parents, this included soft drinks, sweetened juice, sweetened tea, tea or coffee with cream and/or sugar, and energy drinks. For children, this included soda pop and fruit-flavored drinks. Items were grouped to construct variables to assess daily intake of dairy-based beverages. For parents, this included whole, reduced fat, and fat-free milk. For children, this included milk, milk on cereal, and cocoa made with milk.

Parents provided information about demographic characteristics for themselves (age, gender, ethnicity, race, education, employment, and food assistance) and their child (age and gender). Researchers measured height and weight of children using standard procedures [34]. Mean height and weight values were used to calculate body mass index (BMI). Questionnaires were completed on iPads using a Qualtrics survey platform (Qualtrics, Provo, UT, USA). Completion of the research activities took about 10 to 15 min per family. 

### 2.3. Data Analysis

Responses to grouped items (knowledge, availability, and intake) were summed across the items to produce summed scores for these continuous variables. Child daily SSB and dairy beverage intake variables were dichotomized into “8 ounces or more” and “less than 8 ounces” (8 oz = 1 fluid cup or 237 mL). For categorical variables, frequency counts and percentages were calculated. For continuous variables, means and standard deviations were computed. 

Chi square tests were used to identify univariate associations between child intake outcomes (SSB and dairy beverages), child sex, parent sex, food assistance (any/none), number of children in the home, education, and employment (not shown). 

Two multiple logistic regression models were constructed to estimate adjusted odds ratios and 95% confidence intervals for the binary outcomes of child sweetened beverage intake (8 ounces or more/day) and child dairy beverage intake (8 ounces or more/day). Pearson correlation analyses were conducted to determine which potential variables should be included in the regression models. Both models included summed scores for availability of sugar-sweetened beverages and dairy beverages; summed knowledge scores for beverage nutrition, dairy beverages/calcium, and sugar in beverages; parent intake of SSBs (in the dairy beverage intake model only) and dairy beverages (in the SSB intake model only); and child intake of orange juice and dairy beverages, adjusted for child sex and child age. All beverage intake variables were continuous, independent variables in the regression models. Statistical significance was assessed at the *p* = 0.05 level in all analyses. Statistical Analysis System software (SAS; version 9.3, SAS Institute Inc., Cary, NC, USA) was used to analyze all data. 

## 3. Results

### 3.1. Participant Characteristics

Information about parent (*n* = 194) and child (*n* = 194) demographic characteristics and the household is presented in Table 1. The majority of parents were white (92%), had some college/≥4-year degree (94%), were women (81%) and were employed full/part time (82%). Mean age and BMI were 43 years and 26.2 BMI units, respectively. Mean child (SD) age was 10.6 (1.1); 49% were boys.

### 3.2. Home Beverage Availability

Parents reported the availability of a variety of beverages in their home (Table 2). The two beverages that were most commonly always available were milk (whole, low-fat, or soy) (76% of homes) and bottled water (41%). The beverages rated as sometimes available at home were flavored milk (57%), hot chocolate (75%), regular soda (59%), 100% fruit juice (44%), and fruit drinks (57%). Diet soda was rated as never available at home for 44% of the respondents. 

### 3.3. Beverage Intake and Knowledge Scores

SSB and dairy beverage intakes are shown in Table 3. For parents, water was consumed in the highest amount (mean = 28.8 oz/day, not shown), followed by low fat or fat free milk (mean = 9.3 oz/day) (Table 3), and tea or coffee without sweetener (mean = 4 oz/day, not shown) and with sweetener (mean = 5.1 oz/day, not shown). Parent intake of diet soft drinks was about twice that of regular soft drinks (mean = 4.3 oz (not shown) vs. mean = 1.8 oz (Table 3), respectively). Energy and sports drinks, juice drinks, and whole milk were consumed by parents at low levels (mean < 3 oz/day). Parent intake of whole milk (mean = 0.9 oz/day) and reduced fat 2% milk (mean = 2 oz/day) were about one third the amount of fat-free milk (mean = 9.3 oz/day) (Table 3).

Water was also consumed in the highest amount by children (mean = 19 oz/day, not shown), followed by milk (mean = 11 oz/day) (Table 3). No child reported consuming more than 16 oz milk/day. Milk on cereal was about one third of the total amount of milk consumed. Mean intake for regular soda pop and fruit-flavored drinks by children was 2 and 3 oz/day, respectively (Table 3). Children’s intake of tea or coffee was limited (not shown). 

All three knowledge scores were in the center of the range. Mean parent beverage nutrition knowledge score was 4.8 of 8 possible points with a range of 2–7 points. The mean dairy/calcium knowledge was 4.6 of 8 possible points with a range of 0–8 points. The mean parent knowledge about sugar in beverages was 4.2 of 7 possible points with a range of 0–7 points. 

### 3.4. Associations between Child Beverage Intake and Parent and Home-Related Factors

The adjusted odds of child sugar-sweetened beverage consumption of 8 ounces or more per day were 1.48 times higher for each additional level of available sugar-sweetened beverages in the home. The adjusted odds ratio of sugar-sweetened beverage intake of 8 ounces or more per day for boys compared to girls was 3.35. No other factors included in the model were significantly associated with child sugar-sweetened beverage intake (Table 4).

The adjusted odds of child dairy beverage intake of 8 ounces or more per day were 1.46 times higher for each additional unit (score) of parent knowledge about sugar in beverages, 1.06 times higher for each additional unit of parent dairy beverage intake, and 1.34 times higher for each additional level of available dairy beverages in the home (Table 4). No other factors included in the model were significantly associated with child dairy beverage intake (Table 4). 

## 4. Discussion

This study examined relationships between parental and home-related factors and child beverage intakes among a convenience sample of parents and early adolescent children (9–12 years). As hypothesized, availability of sugar-sweetened and dairy beverages was associated with child sugar-sweetened and dairy beverage intakes, respectively. Several studies have shown that education level influences the initiation of behaviors to establish healthy lifestyles [35,36,37]. Studies have suggested that higher education levels contribute to having essential health information, knowledge, skills, values, and psychological control in order to choose behaviors to establish healthy lifestyles [38]. The high education level of parents in the current sample may have contributed to their likelihood of controlling home beverage availability to influence child beverage intake. The majority of parents reported only having SSBs available at home sometimes and child intake of SSBs was low compared to national intake data [7]. Previous studies among children 2.5 to 7 years old [39], 5–10 years old [40], and adolescents aged 11 years [41] also showed that SSB availability was associated with child intake among well-educated parents. 

Findings from the current study also correspond with previous studies indicating that boys had a higher SSB intake than girls [42,43,44,45,46]. Selection of SSBs may currently be considered a less healthy choice by teens based on recent media health promotion efforts regarding the sugar content of beverages [47]. Females may be reacting more favorably to these efforts because they tend to have greater concern about body weight [48] and are more likely to rate food choice behaviors as important compared to males [49,50,51]. 

The associations observed in the current study between the availability of dairy beverages at home and child dairy beverage intake were consistent with several studies in samples of primarily white children and adolescents [21,52,53]. On the other hand, Patrick et al. [54] did not find an association between home availability of dairy beverages and child dairy beverage intake among African-American and Hispanic parents and their preschool children. In another study, nearly half of low-income African-American adolescents (10–14 years) did not meet the daily recommendation for dairy foods [55]. A possible reason for the discrepancy in results may be the high prevalence of lactose intolerance across racial and ethnic groups including African Americans and Hispanics, thus limiting both home availability and intake of dairy beverages [56]. 

In the current study, parent dairy/calcium knowledge was not associated with child dairy beverage intake. A previous cross-sectional study with similar knowledge questions and scores showed that frequency of parenting practices such as encouragement, making calcium-rich foods available, and setting expectations for intake of these foods were associated with greater parent calcium knowledge, but relationships with child intake of calcium-intake foods were not examined [57]. In the current study, parent knowledge about sugar in beverages was associated with greater odds for child dairy beverage intake possibly because knowledge about sugar in beverages made parents aware of the importance of dairy beverage intake for their children as a healthier alternative to SSBs. 

Parent dairy beverage intake, a proxy for role modeling, was associated with child dairy beverage intake in the current study. Similarly, other studies have observed a positive association between parental dairy food/beverage intake and child dairy food/beverage intake [52,53,58,59], emphasizing the importance of parental role modeling as an intervention target to improve child dairy beverage intake. However, such interventions may need to be tailored to parent characteristics such as race, ethnicity and education level.

A strength of the current study was the use of parent-child dyads to better inform relationships between parent knowledge, parenting practices and child behaviors. Another strength was that children reported their own intake rather than dependence on parent report of child behavior. A limitation in this study was the use of a small convenience sample. The convenience sample consisted primarily of white, well-educated parents, likely because data collection was completed at a university-sponsored research building. Therefore, results cannot be applied broadly to other groups of parents. Other limitations are that the difficulty level of the knowledge items was used to develop the knowledge questionnaires, however, further validity testing was not performed. Intake of dairy foods in addition to dairy-based beverages that could help children meet dairy food recommendations were not considered. Only one parent in the family was asked to report beverage intake, however, both parents are likely to model beverage intake for their children. Lastly, this study had a cross-sectional study design, therefore cause and effect cannot be determined.

## 5. Conclusions

Results of the current study indicate that controlling home beverage availability and role modeling by parents may influence child beverage intake, whereas only parent knowledge about sugar in beverages was associated with child dairy beverage intake. Further study to better understand the breadth and type of knowledge topics is needed to inform the development of educational interventions for parents. Interventions for parents that focus on limiting the home availability of sugar-sweetened beverages and role modeling dairy beverage intake may be effective in promoting healthy beverage intakes among children. 

## Figures and Tables

**Table 1:** Demographic characteristics of parent and child participants in a cross-sectional survey.

Parent	Mean (SD) (Range)
Age (*n* = 187)	42.7 (6.1) (30–66)
Body Mass Index (*n* = 143)	26.2 (5.5) (15.8–55.2)
	*n* (%) ^1^
Sex ^1^	
Female	154 (80.6)
Male	37 (19.4)
Relationship to child ^1^	
Parents	188 (97.4)
Other	5 (2.5)
Education ^1^	
High school diploma or GED (General Equivalency Diploma)	11 (5.8)
Some college or technical school	33 (17.3)
4-year college, university degree or advanced degree	147 (77.0)
Employment ^1^	
Homemaker	25 (13.2)
Employed part-time	32 (16.8)
Employed full-time	124 (65.3)
Retired/Not Employed/Student	9 (4.8)
Ethnicity ^1^	
Hispanic or Latino	5 (2.7)
Not Hispanic or Latino	183 (97.3)
Race ^1^	
White or Caucasian	179 (92.3)
Other	12 (6.1)
**Household**	***n* (%) ^1^**
Food Assistance ^1^	
None	170 (87.6)
Public food assistance	20 (10.3)
Children <18 years in the home ^1^	
1 child	30 (15.8)
2–3 children	141 (74.2)
4 or more children	19 (10.0)
Adults >18 years in the home ^1^	
1	21 (11.1)
2	152 (80.0)
3 or more	17 (9.0)
**Children**	**Mean (SD) (Range)**
Age (*n* = 193)	10.6 (1.1) (9–12)
Body Mass Index (*n* = 192)	19.0 (3.4) (13.4–33)
	*n* (%)
Sex ^1^	
Female	98 (50.8%)
Male	95 (49.2%)
Child in home (days/week) ^1^	
1–3	171 (90.0%)
4 or more	19 (10.1%)

^1^ *n* = 188–193 indicating that data are missing from 1–6 parents for these variables.

**Table 2:** Parent-reported frequency of availability of beverages at home in a cross-sectional survey.

How Often Are These Beverages Available in Your Home?	Never	Sometimes	Usually	Always
	*n* (%) ^1^	*n* (%) ^1^	*n* (%) ^1^	*n* (%) ^1^
Regular soda pop ^1^	43 (22.3)	113 (58.6)	22 (11.4)	15 (7.8)
Fruit drinks (any fruit drink flavor, sports drinks, lemonade or sweetened tea) ^1^	27 (14.1)	110 (57.3)	38 (19.8)	17 (8.9)
Whole, 1%, 2% or soy milk ^1^	17 (8.8)	12 (6.2)	17 (8.8)	147 (76.2)
Flavored milk (chocolate, strawberry or other flavors) ^1^	65 (34.2)	108 (56.8)	12 (6.2)	5 (2.6)
Blended yogurt and juice drink or yogurt drink ^1^	77 (39.9)	82 (42.5)	24 (12.4)	10 (5.2)
Hot chocolate, prepared ^1^	35 (18.2)	144 (75.0)	9 (4.7)	4 (2.1)
Diet soda pop ^1^	85 (44.3)	55 (28.7)	25 (13.0)	27 (14.1)
100% fruit juice ^1^	14 (7.35)	85 (44.0)	67 (34.7)	27 (14.0)
Bottled water ^1^	30 (15.5)	43 (22.3)	41 (21.2)	79 (40.9)

^1^ *n* = 190–193 indicating data are missing from 1–4 parents for each variable.

**Table 3:** Self-reported beverage intake of parents and children in a cross-sectional survey based on frequency and amount consumed.

Parents (*n* = 194)	Mean (SD) oz/day	Range
Sugar-sweetened beverage (SSB) intake (5 items)	9.3 (12.6)	0–77
Soft drinks, regular	1.8 (3.9)	0–24
Sweetened juice drink (fruit ades, lemonade, punch, etc.)	1.5 (4.7)	0–48
Sweetened tea	0.6 (2.1)	0–16
Tea or coffee with cream and/or sugar	4.5 (8.4)	0–60
Energy and sports drinks (Red Bull, Gatorade, etc.)	1 (3.2)	0–32
Dairy beverage intake (3 items)	12.2 (13.7)	0–61
Whole milk	0.9 (3.0)	0–24
Reduced fat milk (2%)	2 (5.7)	0–32
Low fat/fat free milk (skim, 1%, buttermilk, soymilk)	9.3 (13.5)	0–60
**Children (*n* = 194)**	**Mean (SD) oz/Day**	**Range**
SSB intake (2 items)	4.8 (6.6)	0.4–48
Soda pop	2.0 (3.5)	0.2–24
Fruit-flavored drinks (lemonades, Kool-Aid, etc.)	2.8 (4.6)	0.2–24
Dairy beverage intake (3 items)	9.3 (2.6)	3–16
Milk (white or chocolate)	10.5 (9.2)	0.1–32
Milk on cereal	2.9 (3.1)	0.1–14
Cocoa made with milk	0.4 (1.4)	0–16

**Table 4:** Associations between child SSB and dairy beverage intakes and parent and home-related factors based on multiple logistic regression models.

Outcome Measure	Child SSB Intake ^1^	*p* Value	Child Dairy Beverage Intake ^1^	*p* Value
	Odds Ratio (95% CI)		Odds Ratio (95% CI)	
Home characteristics				
Availability of SSBs	1.48 (1.03–2.13)	0.03	0.74 (0.53–1.05)	0.09
Availability of dairy beverages	1.27 (0.92–1.77)	0.15	1.34 (1.03–1.73)	0.03
Parent characteristics				
Beverage nutrition knowledge	0.90 (0.55–1.34)	0.50	0.76 (0.52–1.13)	0.17
Sugar in beverages knowledge	0.83 (0.58–1.20)	0.32	1.46 (1.06–1.99)	0.02
Dairy/calcium knowledge	1.07 (0.81–1.43)	0.63	0.96 (0.75–1.22)	0.72
SSB intake	0.99 (0.95–1.03)	0.59	1.00 (0.96–1.03)	0.91
Parent dairy beverage intake	0.98 (0.95–1.02)	0.38	1.06 (1.02–1.10)	0.01
Child characteristics				
Sex (boy vs. girl)	3.35 (1.30–8.66)	0.01	1.49 (0.69–3.26)	0.31
Orange juice intake	1.11 (0.97–1.27)	0.14	1.15 (0.98–1.34)	0.09
Dairy beverage intake	1.09 (0.91–1.30)	0.35	-	-
SSB intake	-	-	1.00 (0.94–1.06)	0.91

^1^ Odds ratios are adjusted for child sex and age and other factors in the model, *n* = 194.

## Supplementary Materials

The following are available online at www.mdpi.com/1660-4601/14/8/929/s1, Beverage nutrition knowledge (8 items), Dairy/calcium knowledge (8 items) and Knowledge about sugar in beverages (7 items).

## References

[1] U.S. Department of Health and Human Services and U.S. Department of Agriculture (2015). 2015–2020 Dietary Guidelines for Americans. 8th Edition. http://health.gov/dietaryguidelines/2015/guidelines/.

[2] Dror D.K., Allen L.H. (2014). Dairy product intake in children and adolescents in developed countries: Trends, nutritional contribution, and a review of association with health outcomes. Nutr. Rev..

[3] De Ruyter J.C., Olthof M.R., Seidell J.C., Katan M.B. (2012). A trial of sugar-free or sugar-sweetened beverages and body weight in children. N. Engl. J. Med..

[4] Malik V.S.V., Pan A., Willett W.C., Hu F.B.F. (2013). Sugar-sweetened beverages and weight gain in children and adults: A systematic review and meta-analysis. Am. J. Clin. Nutr..

[5] Wang M., Yu M., Fang L., Hu R.-Y. (2015). Association between sugar-sweetened beverages and type 2 diabetes: A meta-analysis. J. Diabetes Investig..

[6] Ambrosini G.L., Oddy W.H., Huang R.C., Mori T.A., Beilin L.J., Jebb S.A. (2013). Prospective associations between sugar-sweetened beverage intakes and cardiometabolic risk factors in adolescents. Am. J. Clin. Nutr..

[7] Rosinger A., Herrick K., Gahche J., Park S. (2017). Sugar-sweetened beverage consumption among U.S. youth, 2011–2014. NCHS Data Brief.

[8] LaRowe T., Moeller S., Adams A. (2007). Beverage patterns, diet quality, and body mass index of U.S. preschool and school-aged children. J. Am. Diet. Assoc..

[9] Piernas C., Mendez M.A., Ng S.W., Gordon-Larsen P., Popkin B.M. (2014). Low-calorie- and calorie-sweetened beverages: Diet quality, food intake, and purchase patterns of U.S. household consumers. Am. J. Clin. Nutr..

[10] Vartanian L.R., Schwartz M.B., Brownell K.D. (2007). Effects of soft drink consumption on nutrition and health: A systematic review and meta-analysis. Am. J. Public Health.

[11] Quann E., Fulgoni V., Auestad N. (2015). Consuming the daily recommended amounts of dairy products would reduce the prevalence of inadequate micronutrient intakes in the United States: Diet modeling study based on NHANES 2007–2010. Nutr. J..

[12] Miller G., Merlo C., Demissie Z., Sliwa S., Park S. (2017). Trends in beverage consumption among high school students—United States, 2007–2015. MMWR Morb. Mortal. Wkly. Rep..

[13] Bandura A. (1986). Social Foundations of Thought and Action: A Social Cognitive Perspective.

[14] Santiago-Torres M., Adams A., Carrel A. (2014). Home food availability, parental dietary intake, and familial eating habits influence the diet quality of urban Hispanic children. Child. Obes..

[15] Wyse R., Campbell E., Nathan N. (2011). Associations between characteristics of the home food environment and fruit and vegetable intake in preschool children: A cross-sectional study. BMC Public Health.

[16] Vaughn A.E., Dearth-Wesley T., Tabak R.G., Bryant M., Ward D.S. (2017). Development of a comprehensive assessment of food parenting practices: The home self-administered tool for environmental assessment of activity and diet family food practices survey. J. Acad. Nutr. Diet..

[17] Yee A.Z.H., Lwin M.O., Ho S.S. (2017). The influence of parental practices on child promotive and preventive food consumption behaviors: A systematic review and meta-analysis. Int. J. Behav. Nutr. Phys. Act..

[18] Diamant A., Babey S., Jones M. (2009). Teen Dietary Habits Related to Those of Parents. UCLA Health Policy Brief.

[19] Reicks M., Degeneffe D., Ghosh K., Bruhn C., Goodell L.S., Gunther C., Auld G., Ballejos M., Boushey C., Cluskey M. (2012). Parent calcium-rich-food practices/perceptions are associated with calcium intake among parents and their early adolescent children. Public Health Nutr..

[20] Bauer K.W., Neumark-Sztainer D., Fulkerson J.A., Hannan P.J., Story M. (2011). Familial correlates of adolescent girls’ physical activity, television use, dietary intake, weight, and body composition. Int. J. Behav. Nutr. Phys. Act..

[21] Larson N.I., Story M., Wall M., Neumark-Sztainer D. (2006). Calcium and dairy intakes of adolescents are associated with their home environment, taste preferences, personal health beliefs, and meal patterns. J. Am. Diet. Assoc..

[22] Bere E., Sørli Glomnes E., te Velde S.J., Klepp K.-I. (2008). Determinants of adolescents’ soft drink consumption. Public Health Nutr..

[23] De Craemer M., De Decker E., De Bourdeaudhuij I., Vereecken C., Deforche B., Manios Y., Cardon G. (2012). Correlates of energy balance-related behaviours in preschool children: A systematic review. Obes. Rev..

[24] Park S., Onufrak S., Sherry B., Blanck H. (2014). The relationship between health-related knowledge and sugar-sweetened beverage intake among U.S. adults. J. Acad. Nutr. Diet..

[25] Bjelland M., Hausken S.E.S., Sleddens E.F.C., Andersen L.F., Lie H.C., Finset A., Maes L., Melbye E.L., Glavin K., Hanssen-Bauer M.W. (2014). Development of family and dietary habits questionnaires: The assessment of family processes, dietary habits and adolescents’ impulsiveness in Norwegian adolescents and their parents. Int. J. Behav. Nutr. Phys. Act..

[26] Munsell C., Harris J.L., Sarda V., Schwartz M.B. (2016). Parents’ beliefs about the healthfulness of sugary drink options: Opportunities to address misperceptions. Public Health Nutr..

[27] Cluskey M., Auld G., Edlefsen M., Zaghloul S., Bock M., Bourshey C.J., Bruhn C., Goldberg D., Misner S., Olson B. Calcium Knowledge, Concern, and Expectations for Intake among Parents of Asian, Hispanic and Non-Hispanic White Early Adolescents. http://ncsu.edu/ffci/publications/2008/v13-n3-2008-winter/index-v13-n3-winter-2008.php.

[28] U.S. Department of Agriculture, Agricultural Research Service (2011). USDA National Nutrient Database for Standard Reference, Release 24. http://www.ars.usda.gov/ba/bhnrc/ndl.

[29] National Recreation and Park Association (2004). Hearts n' Parks—Report of 2004 Magnet Center Performance Data.

[30] Ross A.C., Taylor C.L., Yaktine A.L., Del Valle H.B. (2010). Dietary Reference Intakes for Calcium and Vitamin D.

[31] Neumark-Sztainer D., Wall M., Perry C., Story M. (2003). Correlates of fruit and vegetable intake among adolescents: Findings from Project EAT. Prev. Med. (Baltim).

[32] Hedrick V., Savla J., Comber D., Flack K. (2012). Development of a brief questionnaire to assess habitual beverage intake (BEVQ-15): Sugar-sweetened beverages and total beverage energy intake. J. Acad. Nutr. Diet..

[33] Rockett H., Berkey C., Field A., Colditz G. (2001). Cross-sectional measurement of nutrient intake among adolescents in 1996. Prev. Med. (Baltim).

[34] Lohman T., Roache A., Martorell R. (1992). Anthropometric standardization reference manual. Med. Sci. Sports Exerc..

[35] Campbell K., Abbott G., Spence A., Crawford D. Home Food Availability Mediates Associations between Mothers’ Nutrition Knowledge and Child Diet. http://www.sciencedirect.com/science/article/pii/S0195666313003358.

[36] McLeod E., Campbell K., Hesketh K. (2011). Nutrition knowledge: A mediator between socioeconomic position and diet quality in Australian first-time mothers. J. Am. Diet. Assoc..

[37] Vereecken C., Maes L. (2010). Young children’s dietary habits and associations with the mothers’ nutritional knowledge and attitudes. Appetite.

[38] Rosenkranz R. (2008). Model of the home food environment pertaining to childhood obesity. Nutr. Rev..

[39] De Coen V., Vansteelandt S., Maes L., Huybrechts I., De Bourdeaudhuij I., Vereecken C. (2012). Parental socioeconomic status and soft drink consumption of the child. The mediating proportion of parenting practices. Appetite.

[40] Kunin-Batson A., Seburg E., Crain A. (2015). Household factors, family behavior patterns, and adherence to dietary and physical activity guidelines among children at risk for obesity. J. Nutr..

[41] Bjelland M., Lien N., Grydeland M., Bergh I.H., Anderssen S.A., Ommundsen Y., Klepp K.I., Andersen L.F. (2011). Intakes and perceived home availability of sugar-sweetened beverages, fruit and vegetables as reported by mothers, fathers and adolescents in the HEIA (HEalth In Adolescents) study. Public Health Nutr..

[42] Park S., Blanck H., Sherry B., Brener N. (2012). Factors associated with sugar-sweetened beverage intake among United States high school students. J. Nutr..

[43] Ogden C.L., Kit B.K., Carroll M.D., Park S. (2011). Consumption of sugar drinks in the United States, 2005–2008. NCHS Data Brief.

[44] Ranjit N., Evans M., Byrd-Williams C., Evans A. (2010). Dietary and activity correlates of sugar-sweetened beverage consumption among adolescents. Pediatrics.

[45] Wang Y.C., Bleich S.N., Gortmaker S.L. (2008). Increasing caloric contribution from sugar-sweetened beverages and 100% fruit juices among U.S. children and adolescents, 1988–2004. Pediatrics.

[46] Wiecha J.L., Finkelstein D., Troped P.J., Fragala M., Peterson K.E. (2006). School vending machine use and fast-food restaurant use are associated with sugar-sweetened beverage intake in youth. J. Am. Diet. Assoc..

[47] Farley T.A., Halper H.S., Carlin A.M., Emmerson E.M., Foster K.N., Fertig F.R. (2017). Mass media campaign to reduce consumption of sugar-sweetened beverages in a rural area of the United States. Am. J. Public Health.

[48] Wardle J., Haase A.M., Steptoe A., Nillapun M., Jonwutiwes K., Bellisie F. (2004). Gender differences in food choice: The contribution of health beliefs and dieting. Ann. Behav. Med..

[49] Courtenay W.H., Mccreary D.R., Merighi J.R. (2002). Gender and ethnic differences in health beliefs and behaviors. J. Health Psychol..

[50] Furnham A., Hrkcaldy B. (1997). Age and sex differences in health beliefs and behaviours. Psychol. Rep..

[51] Wardle J., Steptoe A., Bellisle F., Davou B., Reschke K., Lappalainen R., Fredrikson M. (1997). Healthy dietary practices among European students. Health Psychol..

[52] Fisher J.O., Mitchell D.C., Smiciklas-Wright H., Mannino M.L., Birch L.L. (2004). Meeting calcium recommendations during middle childhood reflects mother-daughter beverage choices and predicts bone mineral status. Am. J. Clin. Nutr..

[53] Hanson N.I., Neumark-Sztainer D., Eisenberg M.E., Story M., Wall M. (2005). Associations between parental report of the home food environment and adolescent intakes of fruits, vegetables and dairy foods. Public Health Nutr..

[54] Patrick H., Nicklas T., Hughes S.O., Morales M. (2005). The benefits of authoritative feeding style: Caregiver feeding styles and children’s food consumption patterns. Appetite.

[55] Wang Y., Jahns L., Tussing-Humphreys L., Xie B., Rockett H., Liang H., Johnson L. (2010). Dietary intake patterns of low-income urban African-American adolescents. J. Am. Diet. Assoc..

[56] Suchy F.J., Brannon P.M., Carpenter T.O., Fernandez J.R., Gilsanz V., Gould J.B., Hall K., Hui S.L., Lupton J., Mennella J. (2010). National Institutes of Health Consensus Development Conference: Lactose intolerance and health. Ann. Intern. Med..

[57] Gunther C.W., Rose A.M., Bruhn C., Cluskey M., Reicks M., Richards R., Wong S.S., Boushey C.J., Misner S., Olson B. (2015). Parents’ calcium knowledge is associated with parental practices to promote calcium intake among parents of early adolescent children. J. Ext..

[58] Lee S., Reicks M. (2003). Environmental and behavioral factors are associated with the calcium intake of low-income adolescent girls. J. Am. Diet. Assoc..

[59] Reicks M., Ballejos M.E., Goodell L.S., Gunther C., Richards R., Wong S.S., Auld G., Boushey C.J., Bruhn C., Cluskey M. (2011). Individual and family correlates of calcium-rich food intake among parents of early adolescent children. J. Am. Diet. Assoc..

