# Supplementary material for: Beverage Intake among Children: Associations with Parent and Home-Related Factors
Source: Int J Environ Res Public Health. 2017 Aug 18;14(8):929. doi: 10.3390/ijerph14080929 (PMC5580631; doi:10.3390/ijerph14080929)
Supplement: Supplementary file 1 [file ijerph-14-00929-s001.pdf]

Beverage nutrition knowledge (8 items)

1. Which is better for your child's health?

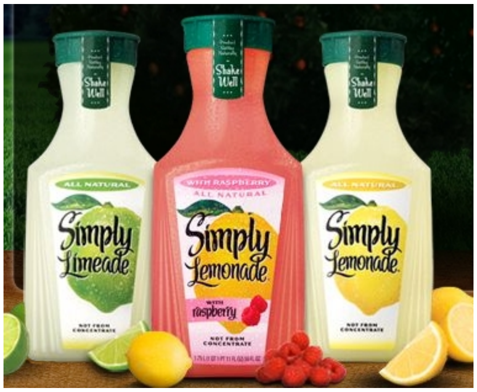

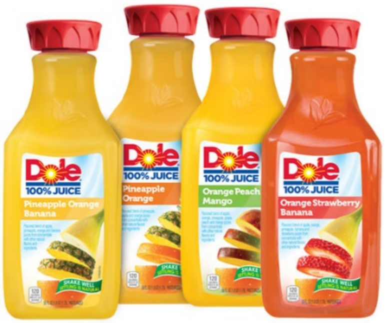

Fruit drinks

100% fruit juice (correct answer)

2. Which is better for your child's health?

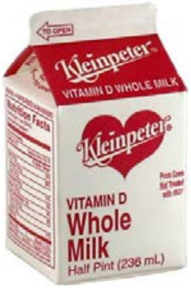

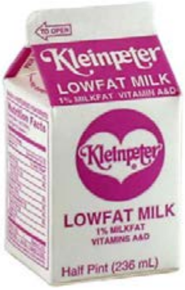

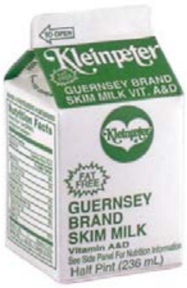

Whole milk

Low-fat milk (correct answer)

3. Which is better for your child's health?

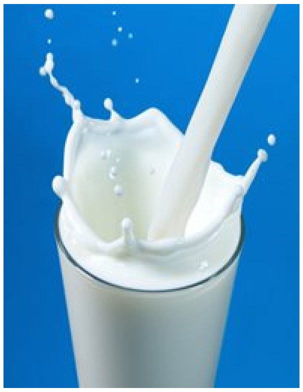

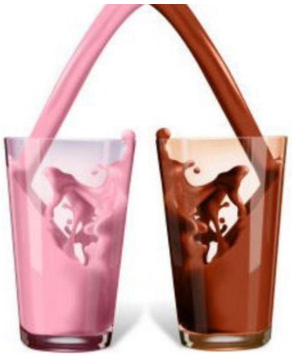

Plain milk (correct answer)

Sweetened flavored milk

4. Which type of milk has the most calories in 1 cup?
    - Whole chocolate milk (correct answer)
    - Whole white milk
    - 2% white milk
    - I do not know (scored as incorrect)

5. Which type of milk has the least calories in 1 cup?
    - 1% white milk
    - Nonfat skim white milk (correct answer)
    - Nonfat skim chocolate milk
    - I do not know (scored as incorrect)

6. Which beverage has the most calories in 1 cup?
    - 100% grape juice, unsweetened (correct answer)
    - Low fat, skim white milk
    - Regular cola, carbonated
    - I do not know (scored as incorrect)

7. About how many calories does a moderately active 9-12 year old boy or girl need each day?
    - 1000-1600 calories
    - 1600-2200 calories (correct answer)
    - 2200-2800 calories
    - 2800-3400 calories
    - I do not know (scored as incorrect)

8. How much water should a child who is 9-12 years-old drink each day?
    - 2-3 cups each day
    - 5-6 cups each day
    - 9-10 cups each day (correct answer)
    - I do not know (scored as incorrect)

Dairy/calcium knowledge (8 items)

Mark true, false, or I do not know (scored as incorrect) for the following questions:

1. 1 cup of calcium-fortified orange juice has about the same amount of calcium as 1 cup of dairy milk. (T)
2. 1 cup of non-dairy milk (soy, rice, or almond) has about the same amount of calcium as 1 cup of dairy milk. (T)
3. 1 cup of lactose free dairy milk (Lactaid™) has about the same amount of calcium as 1 cup of regular dairy milk. (T)
4. Boys need more calcium than girls. (F)
5. Most 9-12 year-old girls are not getting enough calcium. (T)
6. Most 9-12 year-old boys are not getting enough calcium. (T)

7. How much calcium is recommended for children 9-12 years-old each day?
   - 500 milligrams each day
   - 800 milligrams each day
   - 1300 milligrams each day (correct answer)
   - I do not know (scored as incorrect)

8. How many cups of milk would a 9-12 year-old child need to drink each day to get the calcium he/she needs?
   - 1 cup
   - 2 cups
   - 3 cups (correct answer)
   - 4 cups
   - 5 cups
   - I do not know (scored as incorrect)

Knowledge about sugar in beverages (7 items)

Please look at this beverage product to answer the following questions:

For this bottle, list the number of servings, calories per serving, and grams of sugars.

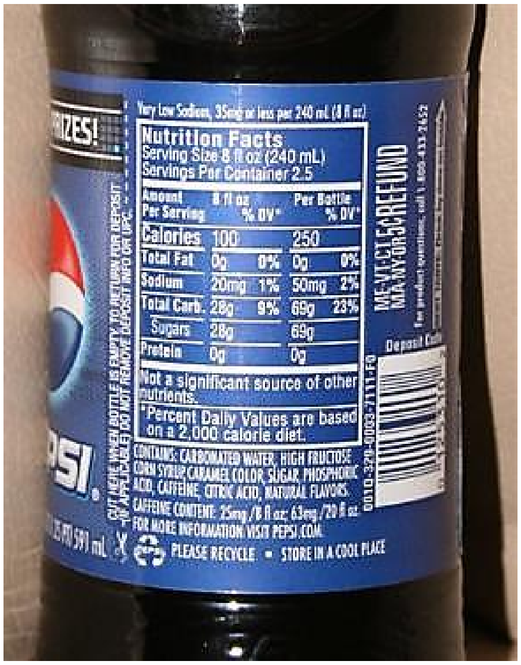

1. Servings: 2.5
2. Calories: 250
3. Grams of sugars: 69

4. How many calories are in 1 teaspoon of sugar?
   - 16 calories (correct answer)
   - 30 calories
   - 45 calories
   - I do not know (scored as incorrect)

5. What is the highest percentage of total calories that should come from sugar for children 9-12 years-old?
   - 15% (correct answer)
   - 30%
   - 45%
   - I do not know (scored as incorrect)

6. How many calories are in a 12 ounce can of regular cola soda pop?
   - About 150 calories (correct answer)
   - About 200 calories
   - About 250 calories
   - I do not know (scored as incorrect)

7. Which beverage has the most sugar in 1 cup?
    - 100% orange juice, unsweetened
    - Canned fruit punch
    - Regular cola, carbonated (correct answer)
    - I do not know (scored as incorrect)
